# Potential formula of the nonregular *m* × *n* fan network and its application

**DOI:** 10.1038/s41598-018-24164-x

**Published:** 2018-04-11

**Authors:** Zhen Tan, Zhi-Zhong Tan, Jianxin Chen

**Affiliations:** 1 School of Electronics and Information, Nantong University, Nantong, 226019 China; 2 Department of physics, Nantong University, Nantong, 226019 China

## Abstract

Potential formula of an arbitrary resistor network has been an unsolved problem for hundreds of years, which is an interdisciplinary problem that involves many areas of natural science. A new progress has been made in this paper, which discovered the potential formula of a nonregular *m* × *n* fan network with two arbitrary boundaries by the Recursion-Transform method with potential parameters (simply call RT-V). The nonregular *m* × *n* fan network is a multipurpose network contains several different types of network model such as the interesting snail network and hart network. In the meantime, we discussed the semi-infinite fan network and a series of novel and special conclusions are produced, the effective resistance is educed naturally. The discovery of potential formulae of resistor network provides new theoretical tools and techniques for related scientific research.

## Introduction

Modelling resistor network to study scientific problem is an important idea, the initial progress of circuit theory dates back to 1845, a German scientist Kirchhoff who proposed the node current law and the circuit voltage law^1^. From then on, the electrical industry has begun to make progress and promote social development, and many problems has been resolved by modelling resistor network by numerous researchers. Nowadays, the circuit networks have been attracting more attention in the recent years since they can be applied to the model both electrical and non-electrical systems involving many sciences problems^2–12^. For example, the calculation of effective resistances involves a wide range of interdisciplinary problems: the problem of classical transport^2^, electromigration phenomena^3^, lattice Greens fusnctions^4,5^, resistance distance^6^ and so on. As is known to all, the mean field theory is widely used to multiple fields, modelling the resistor network can also help to carry on the research of the mean field theory^7^.

In real life, many problems in the field of natural science and physics can be attributed to Laplace equation and Poisson equation^8,9^. Searching for the potential solutions of Laplace’s equation has been an important question involved many fields of science and physics, such as the fields of fluid dynamics, heat conduction, electricity, electromagnetism, astronomy and so on. The solution of Laplace’s equation is subject to boundary conditions, the different boundary conditions seriously affect the solution of Laplace equation. When the boundary geometry is a bit complicated, one must use the computer to resolve the numerical solution, or use the graphical method to draw the equipotential surface or lines of force field. Thus, searching for the exact potential equation of the resistor network become an urgent problem^10^.

We revisit the research history of the network model, it is found that it is usually very difficult to obtain the explicit resistance and potential formulae of the complex networks because the boundary condition is like a trap or wall, which affects the electrical characteristics (current, resistance, potential) of the finite network^10–37^. As this reason that researchers have found several different effective methods to evaluate the effective resistance of resistor network with different structure, but the potential formula of the complex resistor network has always been an unsolved problem for hundreds of years. About the research of resistor network, Cserti^11^ and Giordano^12^ derived the resistance formula of the infinite network by the Green function technique. Wu^13^ formulated a Laplacian matrix method and achieved the exact expressions for the effective resistance in both infinite and finite networks, and the Laplacian approach has also been applied to the complex impedance network^14^. Next refs^15,16^ researched the asymptotic expansion of the resistance between two maximum separated nodes, and Chair^17,18^ researched two resistor networks by the Laplacian matrix method. In 2011 ref.^19^ built a new method to study network model, next Tan, Zhou and Yang proposed a conjecture of cobweb model^20^, shortly after that Izmailian *et al*. improved the Laplacian method and proved the validity of the conjecture^21^, and gave a general resistance formula of the cobweb network, next the globe network^22^ and fan (a rectangle with zero resistor boundary) network^23^ were solved by the Laplacian method. But the Laplacian method is difficult to study the resistor network with arbitrary elements because it is bound by the explicit solution of the matrix with different parameters.

In recent years, Tan^10,19,26–28^ created a desired Recursion-Transform (RT) method in the process of continuous improvement. The superiority of the RT method is that studying resistor network just need one matrix along one directions, which, avoids two Laplacian matries^13,21^, the solution required is just one instead of two eigenvalues, and results given by the RT method is in a single sum. In fact, the RT method have been developed and used to study various types of resistance networks^24–37^. Such as, ref.^24^ studied the resistance of globe network, ref.^25^ computed the resistance of the fan and cobweb networks, refs^26,27^ calculated the resistance of the fan network with arbitrary boundaries, refs^28–30^ calculated the resistance of the cobweb network under different conditions, ref.^31^ studied the resistance of a hammock network by two different methods, ref.^32^ gave out the resistance of the non-regular cylindrical network. refs^33,34^ researched the complex impedance of the rectangular network. ref.^35^ researched the equivalent resistance and impedance of the cylindrical network. Refs^36,37^ researched the complex impedance of the two networks. Recently, ref.^10^ researched the potential functions of the regular fan and cobweb networks by means of the RT method. As a summary, the RT method includes two types, namely *RT-I* method and *RT-V* method, where the *RT-I* method is shorthand for the recursion-transform method with current parameters, and the *RT-V* method is shorthand for the recursion-transform method with potential parameters. The main difference between the two approaches is that it is convenient to calculate the branch currents directly by *RT-I* method, while the *RT-V* method directly calculates the node potential. When using the *RT-I* method to evaluate the potential, it needs to sum the currents such as

$${\rm{\Delta }}{U}_{m\times n}(x,y)=r\sum {I}_{x}^{(i)}$$

Here we are going to derive the potential function of the nonregular fan network by the *RT-V* method pioneered by one of us^10^, and made a new research progress.

**Figure 1:**
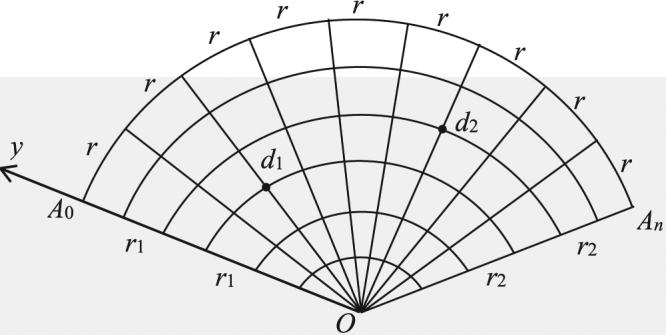
An 6 × 9 fan resistor network with two arbitrary boundaries.

**Figure 2:**
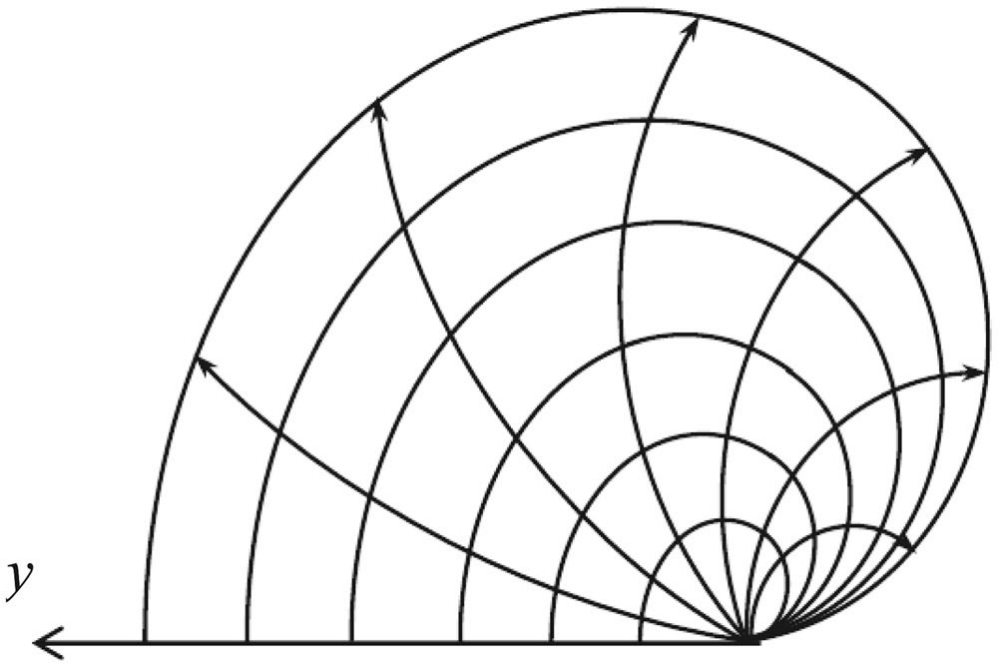
An 6 × 7 snail network of resistors, which is a kind of topological structure of fan network when the resistor on the right edge is zero.

**Figure 3:**
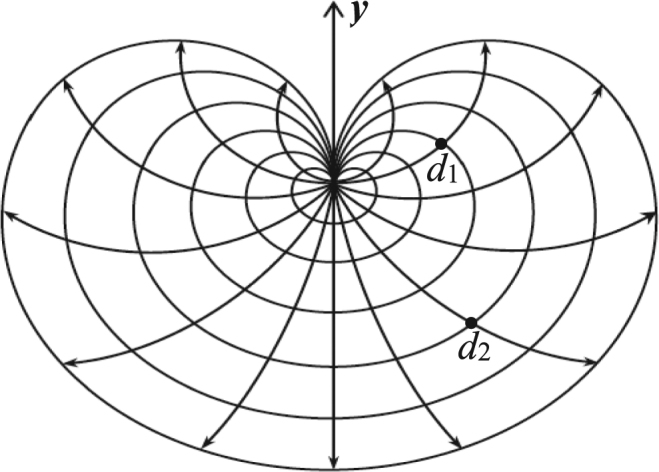
An 6 × 14 hart network of resistors, which is a kind of topological structure of fan network when the resistors on the left and right boundaries are zero.

Figure 1 is called a nonregular fan network, where two arbitrary resistors of *r*_1_ and *r*_2_ are respectively arranged on the left and right boundaries, clearly, two variable resistors represent a variety of network models. Such as, when just *r*_2_ = 0, the fan network degrades into a snail network as shown in Fig. 2, where all nodes on the right edge with zero resistor collapse into a point, when *r*_1_ = *r*_2_ = 0, the fan network degrades into a heart network as shown in Fig. 3, which is an interesting topological structure, where all nodes on the edges with zero resistor collapse into a point. Thus, to achieve potential equation of the nonregular fan network with two arbitrary boundaries is an important physics problem, which can provide a new technique and theory for the related research.

## Results

### Several definitions

In order to simplify the expression of the solutions of matrix equations in the following sections, we define several variables below for later use,

1 $$\begin{array}{rcl}{F}_{k}^{(i)} & = & ({\lambda }_{i}^{k}-{\overline{\lambda }}_{i}^{k})/({\lambda }_{i}-{\overline{\lambda }}_{i}),\,{\rm{\Delta }}{F}_{k}^{(i)}={F}_{k+1}^{(i)}-{F}_{k}^{(i)},\\ {\alpha }_{s,x}^{(i)} & = & {\rm{\Delta }}{F}_{x}^{(i)}+({b}_{s}-1){\rm{\Delta }}{F}_{x-1}^{(i)},{b}_{s}={r}_{s}/{r}_{0}.\end{array}$$

2 $${\beta }_{x,{x}_{s}}^{(i)}=\{\begin{array}{cc}{\alpha }_{1,{x}_{s}}^{(i)}{\alpha }_{2,n-x}^{(i)}\,, & {\rm{if}}\,x\ge {x}_{s}\\ {\alpha }_{1,x}^{(i)}{\alpha }_{2,n-{x}_{s}}^{(i)}\,, & {\rm{if}}\,x\le {x}_{s}\end{array}$$

3 $${G}_{n}^{(i)}={F}_{n+1}^{(i)}+({b}_{1}+{b}_{2}-2){F}_{n}^{(i)}+({b}_{1}-1)({b}_{2}-1){F}_{n-1}^{(i)}.$$

4 $${S}_{k,i}=\,\sin \,({y}_{k}{\theta }_{i}),\,{\theta }_{i}=(2i-1)\pi /(2m+1),$$

5 $$\begin{array}{rcl}{\lambda }_{i} & = & 1+b-b\,\cos \,{\theta }_{i}+\sqrt{{(1+b-b\cos {\theta }_{i})}^{2}-1},\\ {\bar{\lambda }}_{i} & = & 1+b-b\,\cos \,{\theta }_{i}-\sqrt{{(1+b-b\cos {\theta }_{i})}^{2}-1}.\end{array}$$

The above definition is similar to literature 10, 26–30, which is conducive to the unification of physical symbols and the comparison with other results. Such as expressing equation roots by (5), voltage by *U*_*m*×*n*_(*x*, *y*) or ${V}_{x}^{(y)}$, current by *J* or ${I}_{k}^{(i)}$ and so on. The above definitions will be applied in the following all sections, which can make complex results become simple and easy.

### Two general potential formulae

**Figure 4:**
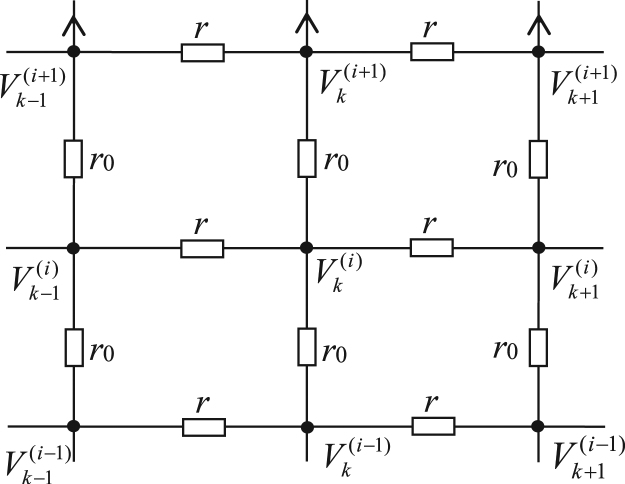
Segment of resistor network with resistor and potential parameters.

Considering a nonregular *m* × *n* fan network as shown in Fig. 1, where two resistors *r*_1_ and *r*_2_ bond on the left and right boundaries, and denote the resistors along the radius and arc directions by *r*_0_ and *r*, and denote the resistor numbers along radius and arc directions by *m* and *n*. Suggesting *O* is the origin of the coordinate system, and the left edge act as Y axis. Denote potential distribution of *d*(*x*, *y*) is shown in Fig. 4, where reads ${U}_{m\times n}(x,y)={V}_{x}^{(y)}$.

We inject current *J* into the lattice at *d*(*x*_1_, *y*_2_) and exit *J* at *d*_2_(*x*_2_, *y*_2_), and select *U*_0_(0, 0) = 0. We find the nodal potential in a nonregular *m* × *n* fan network is

6 $$\frac{{U}_{m\times n}(x,y)}{J}=\frac{2{r}_{0}}{2m+1}\,\sum _{i=1}^{m}(\frac{{\beta }_{{x}_{1},x}^{(i)}{S}_{{y}_{1},i}-{\beta }_{{x}_{2},x}^{(i)}{S}_{{y}_{2},i}}{(1-\,\cos \,{\theta }_{i}){G}_{n}^{(i)}}){S}_{y,i},$$

where ${\beta }_{{x}_{s},{x}_{k}}^{(i)}$, *S*_*k*,*i*_, *θ*_*i*_ and ${G}_{n}^{(i)}$ are, respectively, defined in Eqs (2–4).

When Fig. 1 is a semi-infinite network of *n* → ∞, the nodal potential in an *m* × ∞ resistor network can be written as

7 $$\frac{{U}_{m\times \infty }(x,y)}{J}=\frac{2r}{2m+1}\,\sum _{i=1}^{m}\frac{{\overline{\lambda }}_{i}^{|{x}_{1}-x|}{S}_{1,i}-{\overline{\lambda }}_{i}^{|{x}_{2}-x|}{S}_{2,i}}{\sqrt{{(1-b-b\cos {\theta }_{i})}^{2}-1}}{S}_{y,i}.$$

Formulae (6) and (7) are found for the first time by this paper.

## Method

### *RT-V* method

*RT-V* method is shorthand for the recursion-transform method with potential parameters pioneered by one of us^10^. The *RT-V* method splits the derivation into four parts. The first part creates a main matrix equation of potential distributions along the *Y* axis. The second part derives the constraint equations (including boundary conditions) of nodal potentials. The third part diagonalizes the matrix relation to produce a simple recurrence relation involving only variables on the same *Y* axis, which reduces the problem from two dimensions to one dimension. The fourth part makes the inverse transformation of matrix to derive the exact nodal potential. Here we are going to promote the *RT-V* method to suit to evaluating the nodal potential of the nonregular *m* × *n* fan network. The following is the specific application of the *RT-V* method to derive Eqs (6) and (7).

### Building recursion relations

Assuming {*x*, *y*} is the coordinate of node *d*(*x*, *y*) in the network, and denoting the nodal potential of the fan network is shown in Fig. 4. We express the nodal potential at *d*(*x*, *y*) by $U(x,y)={V}_{x}^{(y)}$, and stipulate *V*_0_ = 0 at the *O*.

Setting up the equations based on the sub-network of Fig. 4. By Kirchhoff law $({\rm{\Sigma }}{r}_{i}^{-1}{V}_{k}=0)$ to set up the node potential equations along the radius direction, we achieve when ignoring the external current source,

8 $$\begin{array}{rcl}{V}_{k+1}^{(1)} & = & (2+2b){V}_{k}^{(1)}-{V}_{k-1}^{(1)}-b{V}_{k}^{(2)},\,i=1\\ {V}_{k+1}^{(i)} & = & (2+2b){V}_{k}^{(i)}-{V}_{k-1}^{(i)}-b{V}_{k}^{(i-1)}-b{V}_{k}^{(i+1)},\,1 < i < m,\\ {V}_{k+1}^{(m)} & = & (2+b){V}_{k}^{(m)}-{V}_{k-1}^{(m)}-b{V}_{k}^{(m-1)},\,i=m\end{array}$$

where *b* = *r*/*r*_0_. We can rewrite Eq. (8) as a matrix form and consider the current *J* flow through network from *d*_1_(*x*_1_, *y*_1_) to *d*_2_(*x*_2_, *y*_2_),

9 $${{\bf{V}}}_{k+1}={{\bf{B}}}_{m}{{\bf{V}}}_{k}-{{\bf{V}}}_{k-1}-r{{\bf{I}}}_{k}{\delta }_{x,k}$$

where ***V***_k_ is an *m* × 1 column matrix,

10 $${{\bf{V}}}_{k}={[{V}_{k}^{(1)},{V}_{k}^{(2)},\cdots ,{V}_{k}^{(m)}]}^{T},$$

and

11 $${I}_{k}^{(i)}=J({\delta }_{{y}_{1},i}-{\delta }_{{y}_{2},i})$$

and **B**_*m*_ is a *m* × *m* tridiagonal matrix,

12 $${{\bf{B}}}_{m}=(\begin{array}{ccccc}(2+2b) & -b & 0 & 0 & 0\\ -b & (2+2b) & -b & 0 & 0\\  & \vdots  & \ddots  & \vdots  & \\ 0 & 0 & -b & (2+2b) & -b\\ 0 & 0 & 0 & -b & (2+b)\end{array}).$$

Next, according to the *RT-V* method^10^ we need to set up the equations of boundary conditions by the left and right edges. Using Kirchhoff’s current law (Σ${r}_{i}^{-1}$*V*_*k*_ = 0) yields

13 $${b}_{1}{{\bf{V}}}_{1}=[{{\bf{B}}}_{m}-(2-{b}_{1}){\bf{E}}]{{\bf{V}}}_{0},$$

14 $${b}_{2}{{\bf{V}}}_{n-1}=[{{\bf{B}}}_{m}-(2-{b}_{2}){\bf{E}}]{{\bf{V}}}_{n},$$

where *b*_*k*_ = *r*_*k*_/*r*_0_, matrix **B**_*m*_ is given by (12).

The above Eqs (9–14) are all equations we need to calculate the potential, we are going to resolve them indirectly by the method of matrix transform.

### Approach of matrix transform

According to the RT method, we obtain after multiplying Eq. (9) from the left-hand side by an *m* × *m* undetermined matrix *Q*_*m*_

15 $${{\bf{Q}}}_{m}{{\bf{V}}}_{k+1}={{\bf{Q}}}_{m}{{\bf{B}}}_{m}{{\bf{V}}}_{k}-{{\bf{Q}}}_{m}{{\bf{V}}}_{k-1}-r{{\bf{Q}}}_{m}{{\bf{I}}}_{k}{\delta }_{x,k}.$$

Evaluating the eigenvalues of matrix **B**_*m*_ by solving determinant equation of det|**B**_*m*_ − *t***E**_*m*_| = 0, we obtain the eigenvalues (*i* = 1, 2, … *m*)

16 $${t}_{i}=2(1+b)-2b\,\cos \,{\theta }_{i},$$

where *θ*_*i*_ = (2*i* − 1)*π*/(2*m* + 1). Next, constructing the matrix transform by the following identity

17 $${{\bf{Q}}}_{m}{{\bf{B}}}_{m}={{\bf{T}}}_{m}{{\bf{Q}}}_{m}.$$

Substituting (16) into (17) yields

18 $${{\bf{Q}}}_{m}=(\begin{array}{cccc}\sin \,{\theta }_{1} & \sin \,2{\theta }_{1} & \cdots  & \sin \,m{\theta }_{1}\\ \sin \,{\theta }_{2} & \sin \,2{\theta }_{2} & \cdots  & \sin \,m{\theta }_{2}\\ \vdots  & \vdots  & \ddots  & \vdots \\ \sin \,{\theta }_{m} & \sin \,2{\theta }_{m} & \cdots  & \sin \,m{\theta }_{m}\end{array}),$$

and we can get the inverse matrix of *Q*_*m*_

19 $${{\bf{Q}}}_{m}^{-1}=\frac{4}{2m+1}(\begin{array}{cccc}\sin \,{\theta }_{1} & \sin \,{\theta }_{1} & \cdots  & \sin \,{\theta }_{m}\\ \sin \,2{\theta }_{1} & \sin \,2{\theta }_{2} & \cdots  & \sin \,2{\theta }_{m}\\ \vdots  & \vdots  & \ddots  & \vdots \\ \sin \,m{\theta }_{1} & \sin \,m{\theta }_{m} & \cdots  & \sin \,m{\theta }_{m}\end{array}).$$

For simplifying our expression, by (15) and (17) we appoint

20 $${{\bf{Q}}}_{m}{{\bf{V}}}_{k}={{\bf{X}}}_{k}\,{\rm{or}}\,{{\bf{V}}}_{k}={({{\bf{Q}}}_{m})}^{-1}{{\bf{X}}}_{k},$$

where **X**_*m*_ is

21 $${{\bf{X}}}_{k}={[{X}_{k}^{(1)},{X}_{k}^{(2)},\cdots ,{X}_{k}^{(m)}]}^{{\rm{T}}},$$

We therefore obtain a main equation after applying (20) and (17) to Eq. (15),

22 $${X}_{k+1}^{(i)}={t}_{i}{X}_{k}^{(i)}-{X}_{k-1}^{(i)}-rJ{\zeta }_{y,i},$$

where

23 $${\zeta }_{1,i}=\,\sin \,{y}_{1}{\theta }_{k},\,{\zeta }_{2,i}=-\,\sin \,{y}_{2}{\theta }_{k}.$$

Analogously, multiplying Eqs (13) and (14) from the left-hand side by matrix *Q*_*m*_ yields

24 $${b}_{1}{X}_{1}^{(i)}=({t}_{i}+{b}_{1}-2){X}_{0}^{(i)},$$

25 $${b}_{2}{X}_{n-1}^{(i)}=({t}_{i}+{b}_{2}-2){X}_{n}^{(i)}.$$

Thus, we obtained all equations to calculating the potential by solving Eqs (22–25).

### General solutions of the matrix equations

Assuming ${\lambda }_{i},{\overline{\lambda }}_{i}$ are the roots of the characteristic equation for Eq. (22), we therefore get Eq. (5). Based on above matrix Eqs (22–25) that we achieve the general solution of ${X}_{k}^{(i)}$ (0 ≤ *k* ≤ *n*),

26 $${X}_{x}^{(i)}=\frac{{\beta }_{x,{x}_{1}}^{(i)}{\zeta }_{1,i}+{\beta }_{x,{x}_{2}}^{(i)}{\zeta }_{2,i}}{({t}_{i}-2){G}_{n}^{(i)}}rJ,$$

where ${\beta }_{x,{x}_{s}}^{(i)}$ is defined in Eq. (2), and *ζ*_1,*i*_, *ζ*_2,*i*_ are given by Eq. (23).

Please note that Eq. (26) is a complex expression which is composed of three piecewise function (0 ≤ *k* ≤ *x*_1_, *x*_1_ ≤ *k* ≤ *x*_2_, *x*_2_ ≤ *k* ≤ *n*).

### Inverse matrix transformation

In order to get the desired potential formula, we make inverse matrix transformation by Eqs (19) and (20), we have

27 $$(\begin{array}{c}{V}_{k}^{(1)}\\ {V}_{k}^{(2)}\\ \vdots \\ {V}_{k}^{(m)}\end{array})=\frac{4}{2m+1}(\begin{array}{cccc}\sin \,{\theta }_{1} & \sin \,{\theta }_{2} & \cdots  & \sin \,{\theta }_{m}\\ \sin \,2{\theta }_{1} & \sin \,2{\theta }_{2} & \cdots  & \sin \,2{\theta }_{m}\\ \vdots  & \vdots  & \ddots  & \vdots \\ \sin \,m{\theta }_{1} & \sin \,m{\theta }_{m} & \cdots  & \sin \,m{\theta }_{m}\end{array})(\begin{array}{c}{X}_{k}^{(1)}\\ {X}_{k}^{(2)}\\ \vdots \\ {X}_{k}^{(m)}\end{array}).$$

By (27), we have

28 $${V}_{k}^{(y)}=\frac{4}{2m+1}\sum _{i=1}^{m}{X}_{k}^{(i)}\,\sin \,(y{\theta }_{i})$$

Putting Eq. (26) into (28), we get

29 $${V}_{k}^{(y)}=J\frac{4r}{2m+1}\sum _{i=1}^{m}\frac{{\beta }_{x,{x}_{1}}^{(i)}{\zeta }_{1,i}+{\beta }_{x,{x}_{2}}^{(i)}{\zeta }_{2,i}}{({t}_{i}-2){G}_{n}^{(i)}}\,\sin \,(y{\theta }_{i}),$$

Putting Eq. (23) into (29), we therefore achieved formula (6).

Proof of Eq. (7). When *n* → ∞ with *m* finite, by Eq. (5) we have ${\lambda }_{i} > 1 > {\bar{\lambda }}_{i} > 0$, together with Eqs (2) and (3), taking limit to the factor of Eq. (6) yields

30 $$\mathop{\mathrm{lim}}\limits_{\begin{array}{l}n\to \infty \\ x\to \infty \end{array}}\frac{{\beta }_{x,{x}_{s}}^{(i)}}{({t}_{i}-2){G}_{n}^{(i)}}=\frac{{\lambda }_{k}^{|{x}_{s}-x|}}{{\lambda }_{i}-{\overline{\lambda }}_{i}}.$$

Substituting (30) into (6), we therefore obtain Eq. (7) after using ${\lambda }_{i}-{\bar{\lambda }}_{i}=2\sqrt{{(1+b-b\cos {\theta }_{i})}^{2}-1}$.

## Discussion

### Applications of the potential formula

In the following applications, we stipulate all parameters are identical with the Eqs (1–5), and all definitions are identical with the preceding part of the text. Especially, the potential in reference nodes *O* satisfies *U*_0_(0,0) = 0. Making use of formulae (6) and (7) we have a series of specific potential formula as follows.

#### Application 1.

Consider a nonregular *m* × *n* fan network with *r*_1_ = *r*_0_ (*r*_2_ is arbitrary) as shown in Fig. 1, the potential of a node *d*(*x*, *y*) in the network is

31 $$\frac{U(x,y)}{J}=\frac{2{r}_{0}}{2m+1}\sum _{i=1}^{m}(\frac{{\beta }_{x,{x}_{1}}^{(i)}{S}_{{y}_{1},i}-{\beta }_{x,{x}_{2}}^{(i)}{S}_{{y}_{2},i}}{(1-\,\cos \,{\theta }_{i})[{F}_{n+1}^{(i)}+({b}_{2}-1){F}_{n}^{(i)}]}){S}_{y,i},$$

where ${\beta }_{{x}_{s},{x}_{k}}^{(i)}$ reduces to ${\beta }_{x,{x}_{s}}^{(i)}={\rm{\Delta }}{F}_{x}^{(i)}{\alpha }_{2,n-{x}_{s}}^{(i)}\,({\rm{if}}\,x\le {x}_{s})$ and ${\beta }_{x,{x}_{s}}^{(i)}={\rm{\Delta }}{F}_{{x}_{s}}^{(i)}{\alpha }_{2,n-x}^{(i)}\,({\rm{if}}\,x\ge {x}_{s})$.

#### Application 2.

Consider a regular *m* × *n* fan resistor network with *r*_1_ = *r*_2_ = *r*_0_ as shown in Fig. 1, the potential of a node *d*(*x*, *y*) in the network is

32 $$\frac{U(x,y)}{J}=\frac{2{r}_{0}}{2m+1}\sum _{i=1}^{m}(\frac{{\beta }_{x,{x}_{1}}^{(i)}{S}_{{y}_{1},i}-{\beta }_{x,{x}_{2}}^{(i)}{S}_{{y}_{2},i}}{(1-\,\cos \,{\theta }_{i}){F}_{n+1}^{(i)}}){S}_{y,i},$$

where ${\beta }_{{x}_{s},{x}_{k}}^{(i)}$ reduces to ${\beta }_{x,{x}_{s}}^{(i)}={\rm{\Delta }}{F}_{x}^{(i)}{\rm{\Delta }}{F}_{n-{x}_{s}}^{(i)}\,({\rm{if}}\,x\le {x}_{s})$ and ${\beta }_{x,{x}_{s}}^{(i)}={\rm{\Delta }}{F}_{{x}_{s}}^{(i)}{\rm{\Delta }}{F}_{n-x}^{(i)}\,({\rm{if}}\,x\ge {x}_{s})$.

Please note that ref.^10^ researched the regular fan network, our formula (32) is completely equivalent to the result of Eq. (6) in ref.^10^, which verify each other’s correctness.

#### Application 3.

When *b*_2_ = 0 (*r*_2_ = 0), Fig. 1 degrades into a snail network as shown in Fig. 2, the potential of a node *d*(*x*, *y*) in the *m* × *n* snail network can be written as

33 $$\frac{U(x,y)}{J}=\frac{4r}{2m+1}\sum _{i=1}^{m}(\frac{{\beta }_{x,{x}_{1}}^{(i)}{S}_{{y}_{1},i}-{\beta }_{x,{x}_{2}}^{(i)}{S}_{{y}_{2},i}}{{\rm{\Delta }}{F}_{n}^{(i)}+({b}_{1}-1){\rm{\Delta }}{F}_{n-1}^{(i)}}){S}_{y,i},$$

where we redefine ${\beta }_{x,{x}_{s}}^{(i)}={\alpha }_{1,x}^{(i)}{F}_{n-{x}_{s}}^{(i)}\,({\rm{if}}\,x\le {x}_{s})$ and ${\beta }_{x,{x}_{s}}^{(i)}={\alpha }_{1,{x}_{s}}^{(i)}{F}_{n-x}^{(i)}\,({\rm{if}}\,x\ge {x}_{s})$.

#### Application 4.

When *r*_1_ = *r*_2_ = 0, Fig. 1 degrades into a heart network as shown in Fig. 3, the potential of a node *d*(*x*, *y*) in the *m* × *n* heart network can be written as

34 $$\frac{U(x,y)}{J}=\frac{4r}{2m+1}\sum _{i=1}^{m}(\frac{{\beta }_{x,{x}_{1}}^{(i)}{S}_{{y}_{1},i}-{\beta }_{x,{x}_{2}}^{(i)}{S}_{{y}_{2},i}}{{F}_{n}^{(i)}}){S}_{y,i},$$

where we redefine ${\beta }_{x,{x}_{s}}^{(i)}={F}_{x}^{(i)}{F}_{n-{x}_{s}}^{(i)}\,({\rm{if}}\,x\le {x}_{s})$ and ${\beta }_{x,{x}_{s}}^{(i)}={F}_{{x}_{s}}^{(i)}{F}_{n-x}^{(i)}\,({\rm{if}}\,x\ge {x}_{s})$.

#### Application 5.

Consider Fig. 1 of an *m* × *n* fan network, when *d*_2_(*x*, *y*) = *O*(0, 0) (the output current *J* is at the point of *O*), the potential of a node *d*(*x*, *y*) is

35 $$\frac{U(x,y)}{J}=\frac{2{r}_{0}}{2m+1}\sum _{i=1}^{m}(\frac{\sin ({y}_{1}{\theta }_{i})\sin \,(y{\theta }_{i})}{(1-\,\cos \,{\theta }_{i}){G}_{n}^{(i)}}){\beta }_{x,{x}_{1}}^{(i)},$$

where ${\beta }_{x,{x}_{s}}^{(i)}$ is defined in (2), and ${S}_{{y}_{2},i}={S}_{0,i}=\,\sin \,({y}_{2}{\theta }_{i})=0$ is used.

#### Application 6.

Consider the input current *J* locate at the left edge, and output current *J* locate at the right edge, the potential of a node *d*(*x*, *y*) in the *m* × *n* fan network is

36 $$\frac{U(x,y)}{J}=\frac{2}{2m+1}\sum _{i=1}^{m}(\frac{{r}_{1}{\alpha }_{2,n-x}^{(i)}{S}_{{y}_{1},i}-{r}_{2}{\alpha }_{1,x}^{(i)}{S}_{{y}_{2},i}}{(1-\,\cos \,{\theta }_{i}){G}_{n}^{(i)}}){S}_{y,i},$$

where ${\alpha }_{k,x}^{(i)}$ is defined in Eq. (1).

#### Application 7.

In Fig. 1, when *x*_2_ = *x*_1_ (the input and output currents locate at the same radius), the potential of a node *d*(*x*, *y*) in the *m* × *n* fan network is

37 $$\frac{U(x,y)}{J}=\frac{2{r}_{0}}{2m+1}\sum _{i=1}^{m}\frac{({S}_{{y}_{1},i}-{S}_{{y}_{2},i}){S}_{y,i}}{(1-\,\cos \,{\theta }_{i}){G}_{n}^{(i)}}{\beta }_{x,{x}_{1}}^{(i)},$$

where *S*_*k*,*i*_ is defined in Eq. (4), and ${\beta }_{x,{x}_{k}}^{(i)}$ is defined in Eq. (2).

#### Application 8.

In Fig. 1, when *y*_2_ = *y*_1_ (the input and output current locate at the same arc), the potential of a node *d*(*x*, *y*) in the *m* × *n* fan network is

38 $$\frac{U(x,y)}{J}=\frac{2{r}_{0}}{2m+1}\sum _{i=1}^{m}\frac{({\beta }_{x,{x}_{1}}^{(i)}-{\beta }_{x,{x}_{2}}^{(i)}){S}_{{y}_{1},i}{S}_{y,i}}{(1-\,\cos \,{\theta }_{i}){G}_{n}^{(i)}}.$$

#### Application 9.

Assuming Fig. 1 is a semi-infinite ∞ × *n* network, and *m* → ∞ but *n* is finite, and (*x*_1_, *y*_1_) and (*x*, *y*) are finite. When *d*_2_(*x*_2_, *y*_2_)=*O*(0, 0), taking limit *m* → ∞ to Eq. (35), we achieve the potential of a node *d*(*x*, *y*) in a semi-infinite ∞ × *n* network

39 $$\frac{{U}_{\infty \times n}(x,y)}{J}=\frac{{r}_{0}}{\pi }{\int }_{0}^{\pi }\frac{\sin \,({y}_{1}\theta )\sin \,(y\theta )}{1-\,\cos \,\theta }(\frac{{\beta }_{x,{x}_{1}}}{{G}_{n}})d\theta .$$

#### Application 10.

When *d*_2_(*x*_2_, *y*_2_) = *O*(0, 0), *m*, *n* → ∞, but (*x*, *y*) and (*x*_1_, *y*_1_) are finite (means the lattice is finite in the left and bottom, but it is infinite in the right and top), taking limit *n* → ∞ to Eq. (39) together with Eq. (30), we obtain

40 $$\frac{{U}_{\infty \times \infty }(x,y)}{J}=\frac{r}{\pi }{\int }_{0}^{\pi }\frac{{\bar{\lambda }}_{\theta }^{|{x}_{1}-x|}\,\sin ({y}_{1}\theta )\sin \,(y\theta )}{\sqrt{{(1+b-b\cos \theta )}^{2}-1}}d\theta ,$$

where $${\bar{\lambda }}_{\theta }=1+b-b\,\cos \,\theta -\sqrt{{(1+b-b\cos \theta )}^{2}-1}$$.

#### Application 11.

Consider a nonregular *m* × *n* fan network with two arbitrary boundaries as shown in Fig. 1, deriving the effective resistance between *d*_1_(*x*_1_, *y*_1_) and *d*_2_(*x*_2_, *y*_2_) based on *R*_*m*×*n*_(*d*_1_, *d*_2_) = (*U*_1_ − *U*_2_)/*J* by Eq. (6), we get

41 $${R}_{m\times n}({d}_{1},{d}_{2})=\frac{2{r}_{0}}{2m+1}\sum _{i=1}^{m}\frac{{\beta }_{1,1}^{(i)}{S}_{{y}_{1},i}^{2}-2{\beta }_{1,2}^{(i)}{S}_{{y}_{2},i}{S}_{{y}_{1},i}+{\beta }_{2,2}^{(i)}{S}_{{y}_{2},i}^{2}}{(1-\,\cos \,{\theta }_{i}){G}_{n}^{(i)}},$$

where ${\beta }_{1,2}^{(i)}={\alpha }_{1,{x}_{1}}^{(i)}{\alpha }_{2,n-{x}_{2}}^{(i)}$(simply reads ${\beta }_{{x}_{1},{x}_{2}}^{(i)}={\beta }_{1,2}^{(i)}$).

Please note that ref.^27^ has researched the effective resistance of the nonregular fan network based on the branch current parameters, but formula (41) derived here is based on the potential parameters. However, the two results are the same in form even though they used two different methods of calculation. Eq. (41) is a general resistance formula of a nonregular fan network, by Eq. (41), we have a specific result as follows.

#### Application 12.

When *b*_1_ = *b*_2_ = 1 (*r*_1_ = *r*_2_ = *r*_0_), Fig. 1 degrades into an regular *m* × *n* fan network, from (41), we have the effective resistance between *d*_1_(*x*_1_, *y*_1_) and *d*_2_(*x*_2_, *y*_2_) in the regular *m* × *n* fan network

42 $${R}_{m\times n}({d}_{1},{d}_{2})=\frac{2{r}_{0}}{2m+1}\sum _{i=1}^{m}\frac{{\beta }_{1,1}^{(i)}{S}_{{y}_{1},i}^{2}-2{\beta }_{1,2}^{(i)}{S}_{{y}_{2},i}{S}_{{y}_{1},i}+{\beta }_{2,2}^{(i)}{S}_{{y}_{2},i}^{2}}{(1-\,\cos \,{\theta }_{i}){F}_{n+1}^{(i)}},$$

where ${\beta }_{{x}_{k},{x}_{s}}^{(i)}$ reduces to ${\beta }_{k,s}^{(i)}={\rm{\Delta }}{F}_{{x}_{k}}^{(i)}{\rm{\Delta }}{F}_{n-{x}_{s}}^{(i)}$.

Please note that refs^23,25^ have researched the resistance formula of the regular fan network, our formula (42) under the case of *r*_1_ = *r*_2_ = *r*_0_ is completely equivalent to the results of refs^23,25^. This comparison demonstrate the validity of the each other’s conclusion.

Searching for the explicit solutions of the potential function in a complex resistor network is important but difficult. This paper makes a new progress in the study of potential function of the nonregular fan network by using the *RT-V* method for the first time. This means that we obtain the analytical solution of the Poisson equation in a variety of boundary conditions because Poisson equation can simulated by resistor network model.

As applications of the RT method, we obtained a universal potential equation of a nonregular *m* × *n* fan resistor network such as Eq. (6). Obviously, it is easy for us to derive the effective resistance by potential formula such as Eq. (41). As applications of formula (6) that many novel results are produced, such as the interesting results of Eqs (31–38), and the potential formulae of semi-infinite network are produced, such as Eqs (39–40).
